# Induction of antigen spread after sipuleucel-T treatment and its association with improved clinical outcome

**DOI:** 10.1186/2051-1426-1-S1-P101

**Published:** 2013-11-07

**Authors:** Debraj GuhaThakurta, Li-Qun Fan, Tuyen Vu, Nadeem A Sheikh, James B Trager

**Affiliations:** 1 Research Department, Dendreon Corporation, Seattle, WA, USA

## Introduction

An effective cancer immunotherapy that triggers immune-mediated lysis of tumor cells, may lead to the priming of T or B lymphocytes against tumor antigens distinct from the initial target/s of the therapy. This is referred to as antigen or epitope spread. Evidence of antigen spread after treatment may not only provide insights into the mechanism of action (MoA) of cancer immunotherapies, but also provide pharmacodynamic (post-treatment) biomarkers of clinical response and outcome. Sipuleucel-T, an FDA approved autologous cellular immunotherapy for the treatment of symptomatic or minimally symptomatic, metastatic castrate-resistant prostate cancer (mCRPC), is designed to elicit immune responses to the prostate-specific antigen, Prostatic Acid Phosphatase (PAP). We sought to determine if antigen spread occurs in response to sipuleucel-T treatment.

## Study & methods

Using protein microarrays (ProtoArrays®), followed by independent technical validation using Luminex, we evaluated humoral antigen spread after sipuleucel-T treatment in IMPACT, a controlled phase 3 clinical study, where sipuleucel-T has shown to improve overall survival (OS) in mCRPC patients (N Engl J Med, 2010, 363, p. 411). We have also assessed antigen spread in an independent clinical study and evaluated the association of antigen spread with OS in sipuleucel-T treated subjects.

## Results

Subjects in the sipuleucel-T arm of IMPACT showed significant IgG responses on the ProtoArray against targets aberrantly expressed in prostate tumors and targets in pathways involved in prostate cancer progression (including known oncogenes). Control arm subjects did not show significant IgG responses on the ProtoArray. IgG responses to specific antigens identified using the ProtoArray in the sipuleucel-T treated subjects were subsequently validated using Luminex xMAP. This confirmed that the sipuleucel-T treated subjects consistently mounted elevated IgG responses against multiple cancer antigens (other than PAP) after treatment (> 25% subjects showing ≥ 3 fold increase in IgG levels), whereas control subjects did not (≤ 5% subjects showing ≥ 3 fold increase in IgG levels). The IgG responses identified from IMPACT were observed in an independent sipuleucel-T phase 2 clinical study (ProACT, NCT00715078). Additionally, humoral antigen spread was observed to be significantly associated with improved OS in IMPACT.

## Conclusions

This study provides further insight into the MoA of sipuleucel-T, and may assist in the identification of post-treatment biomarkers of clinical outcome. The methods and results presented here may also benefit the development of pharmacodynamic biomarkers of clinical outcome for other cancer immunotherapies.

